# Assessment of the centre of pressure pattern and moments about S2 in scoliotic subjects during normal walking

**DOI:** 10.1186/1748-7161-3-10

**Published:** 2008-08-12

**Authors:** Nachiappan Chockalingam, Surendra Bandi, Aziz Rahmatalla, Peter H Dangerfield, El-Nasri Ahmed

**Affiliations:** 1 Faculty of Health, Staffordshire University, Stoke on Trent, ST4 2DF, UK; 2 Departments of Clinical Anatomy and Cell Biology and Musculo Skeletal Medicine, University of Liverpool, Liverpool, L69 3GE, UK; 3 Hartshill Orthopaedic Unit, University Hospital of North Staffordshire, Stoke on Trent, ST4 6QG, UK

## Abstract

**Background Context:**

Research employing gait measurements indicate asymmetries in ground reaction forces and suggest relationships between these asymmetries, neurological dysfunction and spinal deformity. Although, studies have documented the use of centre of pressure (CoP) and net joint moments in gait assessment and have assessed centre of mass (CoM)-CoP distance relationships in clinical conditions, there is a paucity of information relating to the moments about CoM. It is commonly considered that CoM is situated around S2 vertebra in normal upright posture and hence this study uses S2 vertebral prominence as reference point relative to CoM.

**Purpose:**

To assess and establish asymmetry in the CoP pattern and moments about S2 vertebral prominence during level walking and its relationship to spinal deformity in adolescents with scoliosis.

**Patient sample:**

Nine Adolescent Idiopathic Scoliosis subjects (8 females and 1 male with varying curve magnitudes and laterality) scheduled for surgery within 2–3 days after data collection, took part in this study.

**Outcome measures:**

Kinetic and Kinematic Gait assessment was performed with an aim to estimate the CoP displacement and the moments generated by the ground reaction force about the S2 vertebral prominence during left and right stance during normal walking.

**Methods:**

The study employed a strain gauge force platform to estimate the medio-lateral and anterior-posterior displacement of COP and a six camera motion analysis system to track the reflective markers to assess the kinematics. The data were recorded simultaneously.

**Results:**

Results indicate wide variations in the medio lateral direction CoP, which could be related to the laterality of both the main and compensation curves. This variation is not evident in the anterior-posterior direction. Similar results were recorded for moments about S2 vertebral prominence. Subjects with higher left compensation curve had greater displacement to the left.

**Conclusion:**

Although further longitudinal studies are needed, results indicate that the variables identified in this study are applicable to initial screening and surgical evaluation of scoliosis.

## Introduction

Bipedal locomotion creates a major challenge to our balance control system both in walking and running and it is entirely different to the task of maintaining balance during standing. *Posture*, an angular measure from the vertical, is defined as the description of the orientation of any body segment relative to the gravitational vector (line of action of the ground reaction force) and *balance *describes the dynamics of body posture to prevent falling. During major part of normal walking, body weight is supported by one limb (stance phase) and this part of gait demonstrates several capabilities such as muscular coordination, balance, strength and joint kinematics [1]. Hence impairment to effective propulsion and balance can be identified by examining this phase.

While Centre of Mass (CoM) is a point equivalent of the total body mass in the global reference system, Centre of pressure (CoP) is the point of location of the vertical ground reaction force vector [2]. When both feet are in contact with the ground, the location of CoP under each foot reflects the neural control of the ankle muscles. CoP moves to the anterior with the increased activity of the plantar flexors and it moves laterally with the increase in invertor muscles activity [2].

Although previous investigations indicate that force platforms provide good measurements to calculate the static balance of individuals [3-6], there is a paucity of information on the dynamic balance during walking. More recently, MacWilliams et al. [7] attempted to document the foot kinematics and kinetics during adolescent gait concentrating on foot joint angles, moment and power using normal subjects Another study investigating the CoP and its relationship to foot pathology, indicated how CoP coordinates can be used to calculate the moments about the joint axis of the foot [8]. Previous studies have also indicated the use of CoP to estimate an index to evaluate the function of foot orthoses during walking [9]. More recently, Sloss [10] while studying the effect of foot orthoses on the ground reaction forces, indicated the usefulness of the estimation of CoP displacement during walking and showed a difference of approximately 1 cm at both heel strike and push off peaks.

While a previous study investigated the asymmetries in kinetic gait parameters in scoliotic subjects [11], one of the purposes of this observational study is to determine whether or not it is possible to detect changes in the gait cycle in patients with abnormal spinal curvature using centre of pressure pattern. Although, scoliosis is described as a three dimensional mal-alignment of the vertebral column [12], the aetiology, causation and progression of idiopathic scoliosis remains unclear [13]. Previous studies have indicated a relationship between neuromuscular abnormalities and the aetiology of AIS [14-16].

Lafond et al. [17] compared various methods of human CoM measurement and demonstrated the relationship between CoP and CoM. It was described that CoP oscillates on either side of the CoM, where the CoP displacement always exceeds CoM. Furthermore, the variable CoP-CoM is reported as the error of the postural control system which provides an important insight into the postural control mechanism. While the position CoM can be estimated through various methods [17,18], its relative position varies with changes in the body segments such as limb movements. However, it is commonly accepted to be around S2 vertebra in normal upright posture [19]. A previous investigation while comparing the kinematic and kinetic methods for computing the vertical displacement of CoM during normal walking, indicated that sacral marker method will provide a reasonable approximation of the vertical body CoM displacement in self selected speeds [19]. Studies have indicated that in a static condition the direction of ground reaction force (GRF) vector should point to the location of CoM which in turn projects the CoP. However, in dynamic tasks, since the rate of change of momentum should equal the moment generated by the GRF (which is force times its perpendicular distance relative to the CoM), the direction of GRF vector does not pass through the CoM. This contrary action helps in achieving balance in dynamic conditions [20]. While, balance is generally refered to the preservation of rotational equilibrium, a body's rotation can be assessed by measuring its angular position over time. When there is a change in position over time, the body is said to have angular velocity, which causes either spinning as in gymnastics or falling. Furthermore, someone can fall even if the body's current angular momentum is constant. This fall is caused by a change in angular velocity or angular momentum. This indicates that a rate of change of angular velocity or angular momentum corresponds to a loss of rotational equilibrium.

According to Newton's second law of linear motion, a body's acceleration is determined by the sum of the forces acting on it. Similarly, the Newton-Euler equation indicates that a body's rotational acceleration is determined by the sum of the moments acting on it. If this sum, known as the resultant external moment equals to zero, when measured relative to the body's CoM, then the rate of change of angular momentum equals zero and equilibrium has been achieved. As opposed to a static condition, the GRF does not pass through the CoM in a dynamic situation to achieve this rotational equilibrium.

Although, studies have looked at CoM -CoP distance relationships in clinical conditions [21], there is a paucity of information relating to the moments about CoM. Nault et al. [22] investigated the relationship between standing stability and body posture parameters in AIS using CoP and CoM and indicated that the scoliotic subjects had a decrease in standing stability indicating greater neuromuscular demand. In a simulated gait experiment, Gefen et al. [23] indicated that the medio lateral stability of the foot was characterised by the medio lateral displacement of the centre of pressure.

Another study attempted to correlate the effects of muscle force on the movement of the CoP for increased clinical utility and indicated that the differential CoP movement can be interpreted as a moment arm for the vertical ground reaction force [24].

During normal walking, at the beginning of single stance phase, the centre of pressure lies on the medial-posterior heel. Then it moves through the mid-foot region and continues towards the forefoot, crossing the metatarsal heads to terminate in the region of the great and the second toe. Significant distortions of this pattern can give evidence of abnormal loads on the foot and of problems in the correct progression of the gait [25]. Therefore as scoliosis affects the physical orientation of various body segments [26], the primary aim of this study was to examine the changes in CoP pattern. Since scoliosis is a displacement from normal curvature, it should be indicated in the CoP pattern. In addition, this investigation will aim to estimate the moments about the S2 and its asymmetries during left and right stance during normal walking.

## Methods

The present study employed a strain gauge force platform sized 464 × 508 mm (Advanced Mechanical Technology, Inc, MA, USA) to estimate the medio-lateral and anterior-posterior displacement of CoP, which can be defined as the difference between the maximum and minimum CoP position in either direction throughout the stance phase of the gait cycle. Force platform data were collected simultaneously with kinematic data. The system consisted of a six camera motion analysis system along with APAS and APAS Gait (Ariel Dynamics Inc. USA) software to digitise and analyse the data.

Nine Adolescent Idiopathic Scoliosis subjects (8 females and 1 male) with an average age of 15.33 (SD 2.54) yrs, mass of 50.22 (SD 4.9) kg and average height of 155.55 (SD 8.3) cm) scheduled for surgery within 2–3 days after data collection, took part in this study. Demographic information including the curve level, amplitude (average cobb angle 61 (SD 11.68) degrees) and the laterality was recorded as given in table 1 (The term compensation curve is used for secondary curves). Ethical approval was sought and received from the university and the local health service ethics committees. All subjects were supplied with a written explanation of the study and gave a written consent.

**Table 1:** Demographic information of the subjects

Subject No.	Age	Height(cm)	Weight(kg)	Cobb Angle (erect) (Degrees)	Cobb Level	Side (Convex)	Compensation (Secondary curve)
1	19	177	54	60	T4 – T11	R	Yes, Left Lumbar
2	17	160	56	62	T8 – L2	R	No
3	13	149	49	60	T6 – T12	R	Yes, Min Left Lumbar
4	11	150	40	47	T11 – L3	L	Yes, Min Right thoracic
5	16	159	51	55	T10 – L2	R	No
6	18	155	54	50	T5 – T12	R	Yes, Min Left Lumbar
7	16	155	45	57	T5-T11	R	Yes, Major Left Lumbar
8	14	163	52	85	T6 – T12	R	Yes, Left Lumbar
9	14	159	51	73	T5 – T10	R	Yes, Left Lumbar

All subjects were assessed by an experienced clinician for anthropometric measurements and the subjects had no known lower limb abnormalities including leg length discrepancies. Ground reaction force measurements from left and right foot were made from separate gait trials.

**Figure 1:**
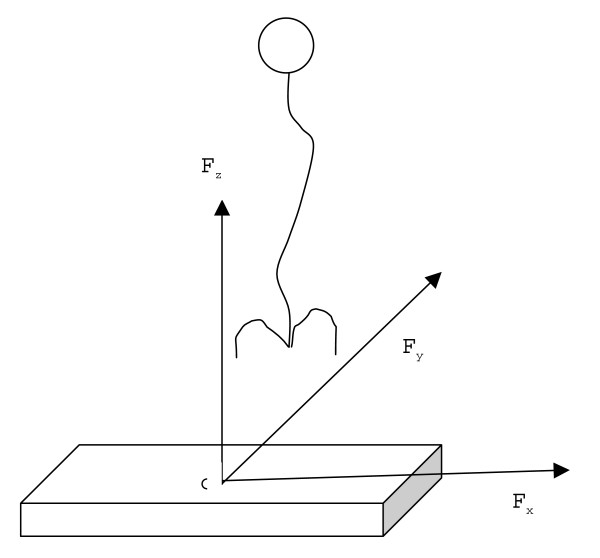
**Axes convention: **o – Geometric Centre of Force plate, in X and Y planes. X – Medio-lateral Direction. Y – Anterio-posterior direction (Direction of walking).

Force platform provided the vertical (Fz), medio lateral (Fx) and anterior/posterior (Fy) components of ground reaction force along with the free moments (Figure 1), Mz, Mx and My. CoP was estimated using the following equations:

CoP_AP _= M_ML_/Fz   and   CoP_ML _= -M_AP_/Fz.

Where M_ML _and M_AP _are the moments around medio-lateral and anterior-posterior components and Fz is the vertical force.

In order to avoid errors during low vertical forces, the maximum and minimum CoP displacement were estimated for 90 percent of the gait cycle ignoring the first and the last five percent of stance phase.

The cameras were calibrated using a standard 1 m^3 ^aluminium cube with 12 markers and they were synchronised using a light emitting diode (LED) device. Force platform information was used to identify heel strike during walking. Markers were placed on vertebral prominences of C7, T6, T12, L4 and S2. This marker placement procedure enabled the creation of 4 segments namely, upper thoracic, lower thoracic, lumbar and sacral. The same researcher positioned the markers every time to avoid inter observer errors and data was collected.

Since it is commonly accepted that the CoM is around S2 vertebra [19], the information from S2 marker was used to estimate the moments about CoM as indicated by the following equation:

Net Moment (M) = Fx (Perpendicular distance (Z) between CoP and S2) + Fz (Perpendicular distance (X) between CoP and S2)

Each participant was then given time to become familiarised with the lab environment and was allowed a number of walking trials prior to data collection. Subjects performed three trials for each foot at the participant's normal walking speed. A valid trial consisted of the participant striking their heel on the force platform without altering their normal gait.

The variables used within this study are maximum and minimum CoP displacement and the moment about the S2 vertebral prominence. Maximum and minimum displacement of CoP is derived from the maximum and minimum CoP coordinates in the medio-lateral and anterio poster axis of the foot. The asymmetry (as indicated by symmetry index – SI) of the right and left legs can be identified using the formula:

$$\text{SI}=\frac{\left(\text{X}1-\text{X}2\right)}{0.5*(\text{X}1+\text{X}2)}*100$$

Where X1 indicates a measure (either CoP displacement or moment) on the right limb and X2 indicates the same measure on the left limb. A symmetry index of 0 indicates that the force parameter is equal on both legs [27].

## Results and Discussion

This observational study has recorded the CoP displacement and the moments about S2 in scoliotic subjects. Table 1 provides demographic information of the subjects. Table 2 provides the maximum displacement of CoP in the medio-lateral direction and anterio-posterior direction, along with the symmetry index. Results indicate a wide variation in the displacement of CoP in the medio lateral direction but not in the antero-posterior direction. This variation could be related to the laterality of both the primary and compensation curves. However, due to small patient numbers and study being observational in nature, it was not possible to test this claim statistically. As shown in the table, a negative SI value indicates displacement to the right. Table 3 illustrates the moments about S2 vertebral prominence. The values are normalised as percentage of height times the body mass of the subject.

**Table 2:** Maximum displacement of CoP in the medio-lateral direction and anterio-posterior direction

	Left Stance		Right Stance		Symmerty Index	
Subject No.	Medio-Lateral (X) m	Anterio-posterior(Y) m	Medio-Lateral (X) m	Anterio-posterior(Y) m	X m	Y m
1	0.016	0.175	0.027	0.172	-53.270	2.057
2	0.015	0.178	0.032	0.165	-70.915	7.712
3	0.034	0.167	0.039	0.160	-14.699	3.984
4	0.020	0.173	0.023	0.173	-12.707	-0.174
5	0.013	0.162	0.017	0.173	-30.490	-6.460
6	0.005	0.132	0.069	0.156	-171.398	-16.289
7	0.027	0.164	0.003	0.167	162.093	-1.993
8	0.023	0.198	0.025	0.201	-7.222	-1.526
9	0.030	0.206	0.035	0.203	-18.058	1.453

**Table 3:** Net Moments about CoM (Normalised as % Height*Body mass)

Subject No.	Left foot (Stance Phase)		Right foot (Stance Phase)		Symmetry Index	
	Max	Min	Max	Min	Max	Min
1	5.054	-0.525	5.544	0.008	-9.248	205.815
2	1.090	-1.828	3.259	-0.111	-99.731	177.002
3	0.007	-5.723	1.098	-3.958	-197.579	36.462
4	1.851	-2.461	2.029	-1.478	-9.172	49.927
5	2.369	-1.484	2.600	-0.368	-9.276	120.430
6	1.229	-1.969	1.439	-3.015	-15.741	-41.993
7	3.630	-1.027	1.190	-2.832	101.223	-93.509
8	0.718	-3.928	4.121	-1.402	-140.632	94.792
9	0.549	-4.263	2.934	-0.867	-136.942	132.388

While reported symmetry indices did not exceed the range reported in previous studies [27], there are marked differences between the left and right sides. A previous study indicated that the subjects with a left compensation curve had a greater SI for a left side impulse and subjects with very little or no compensation had a greater right side impulse [11], this is reflected in the results of CoP displacement reported in the present study for most subjects. Although previous studies indicate lower symmetry for medio-lateral force component [27,16] and the estimation of CoP displacement takes these values into consideration, results do indicate clear differences. Furthermore as indicated by Chockalingam et al. [28] there might be errors due to low vertical forces during the initial contact and push off phases during gait. However, the results in this study consider only the maximum displacement, which occurs during higher vertical forces.

As indicated in previous investigations to achieve balance, the GRF vector points away from the CoM [20] and this is directly reflected in the estimated moments. Higher moment indicates higher displacement from normal to achieve balance during gait. However, due to wide differences in curve magnitude and compensation, this study has not established clear relationship between estimated moments and curve properties. Further longitudinal studies are warranted.

Results demonstrated in this study could be due to gait compensation or could be an indicator of neuro muscular dysfunction. Taking these differences between left and right sides into consideration, this method of kinetic assessment as indicated earlier, could be extended to detect the severity of the curve and gait compensation in scoliotic subjects. However, one of the major limitations of this study is that the reported data is not from successive steps which might lead to variability as indicated by Kim and Eng [16].

Since, scoliosis is defined as a lateral displacement from the normal frontal axis of the body [29] and from the previous investigations [11], centre of pressure is an appropriate measure of the effect of scoliosis. Scoliosis subjects appear to modify their gait in order to compensate for the spinal curvature. While this investigation is a simple observational study and the changes in gait implies a secondary effect, further studies with focussed experimental design are warranted to provide more information on the causative factors. As gait is described as an activity that permits an individual to move from position A to position B while maintaining the body in a generally upright and stable posture [30], it is necessary to assess the gait of subjects to detect abnormalities. Furthermore, gait analysis technologies could be applied to the longitudinal study of children allowing an opportunity for research into the effectiveness of footwear modification designed to alleviate any imbalance during walking. As results indicate a clear difference between the medio-lateral CoP displacement between left and right sides, it can be concluded that this is due to the displacement from normal spinal curvature. However, more in depth longitudinal investigation with varying curve types and magnitudes are required to substantiate this claim.

## Competing interests

The authors declare that they have no competing interests.

## Authors' contributions

NC Contributed substantially to this article. Was involved in study design, data collection, analysis, interpretation and the preparation of manuscript, SB Made a contribution to data analysis and the preparation of manuscript, AR Made a contribution to subject selection, study design, data collection and analysis, PHD Made a contribution to the study design, interpretation of data and preparation of manuscript, ENA Made a contribution to subject selection and interpretation of data.
